# The association between *Helicobacter pylori* infection with overweight/obesity: A protocol for a systematic review and meta-analysis of observational studies

**DOI:** 10.1097/MD.0000000000018703

**Published:** 2020-01-17

**Authors:** Jialiang Chen, Jie Ma, Xinyuan Liu, Shaojie Duan, Ning Liang, Shukun Yao

**Affiliations:** a Graduate School, Beijing University of Chinese Medicine; b Department of Gastroenterology; c Department of Dermatology & Venerology, China-Japan Friendship Hospital; d Center for Evidence-Based Chinese Medicine, Beijing University of Chinese Medicine, Beijing, China.

**Keywords:** BMI, *Helicobacter pylori*, meta-analysis, obesity, systematic review

## Abstract

**Background:**

Obesity has become a serious public health issue. The role of *Helicobacter pylori* (*H. pylori*) infection in overweight or obesity is inconsistent and controversial. It is very necessary to conduct a systematic review and meta-analysis for determining whether *H. pylori* infection was associated with risk of overweight/obesity.

**Methods:**

Four databases **(**PubMed, Web of Science, The Cochrane Library, and EMBASE) will be searched from the inception to January 15, 2019. All observational studies (including cross-sectional, case-control or longitudinal studies) reporting the association between *H. pylori* infection and overweight/obesity will be included. The primary outcome was the presence and incidence of overweight/obesity in conjunction with *H. pylori* infection. Study selection, data extraction, and assessment of quality will be conducted independently by 2 reviewers. RevMan 5.3 and STATA 14.0 software will be used for data synthesis.

**Results:**

The results of this study will provide a better understanding of the role of *H. pylori* infection in overweight/obesity among overall population.

**Conclusion:**

This systematic review and meta-analysis will generate evidence of the association between *H. pylori* infection and overweight/obesity, and the findings of this study will be published in a peer-reviewed journal.

PROSPERO registration number: CRD42019121939

## 1 Introduction

Obesity has emerged as one of the leading public health concerns and has become increasingly common over the past century, with the rapid growth of global economy, the improvement of people's living standards, coupled with unreasonable dietary structure, particularly of high-calorie, delicious foods that are often served in large portions; reducing time spent in physical activities and displacement of sedentary activities such as using electronic devices.^[1,2]^ According to World Health Organization (WHO) classification of body mass index (BMI, kg/m^2^),^[3]^ overweight and obesity is defined as 25 < BMI < 30 and BMI ≥ 30, respectively. A previous systematic review showed that the global prevalence of overweight and obesity combined had risen by 27.5% for adults and 47.1% for children between 1980 and 2013.^[4]^ Meanwhile, the prevalence of overweight and obesity was higher in developed than developing countries.^[4]^ In 2013, the American Medical Association made it clear that obesity should not only be considered a disease risk factor, but also a real disease state.^[5]^ Importantly, obesity shortens life span and affects the function of many organ systems.^[6,7]^ Mortality results from several diseases are also associated with obesity, including diabetes, chronic kidney disease, gastrointestinal disease, and cardiovascular disease.^[8]^

*Helicobacter pylori* (*H. pylori*), a Gram-negative bacterium colonizing the gastric epithelium, can be diagnosed by invasive test of gastric mucosal biopsy or noninvasive tests such as the stool antigen test and urea breath test.^[9]^ A comprehensive meta-analysis recently showed that approximately 4.4 billion individuals in 2015 globally were estimated to be positive for *H. pylori* and the prevalence of *H. pylori* infection varied geographically and is more common in developing countries than in developed ones.^[10]^ Notably, *H. pylori* infection is also a major global public health problem and is recognized as a cofactor in the development of peptic ulcers, gastric cancer, and gastric mucosa-associated lymphoid-tissue lymphoma.^[11]^ Besides, *H. pylori* infection was associated with many extragastric diseases, such as neurological disorders, metabolic syndrome, nonalcoholic fatty liver disease, dermatological diseases, allergy, and respiratory diseases.^[12–16]^

In recent years, there has been increasing interest in the relationship between *H. pylori* infection and obesity.^[17–21]^ One study from a Israel cohort of 235107 individuals indicated *H. pylori* positivity was positively correlated with an increased BMI or overweight/obesity after adjusting for multiple covariates.^[17]^ Similarly, a cross-sectional study in a Chinese population showed a significant positive correlation between BMI and *H. pylori* infection.^[19]^ A recent systematic review showed positive correlation existing between the prevalence of *H. pylori* and obesity in China.^[22]^ However, some studies thought no evidence for a clinically relevant association between *H. pylori* and BMI or obesity.^[20,21]^ Besides, a previous review showed that *H. pylori* infection could be a protective factors of obesity in developed countries.^[23]^ Since the role of *H. pylori* infection in overweight or obesity is inconsistent and controversial, it remains unclear whether *H. pylori* infection is associated with increased risk of overweight or obesity among individuals from different regions. Therefore, it is of great importance to perform a systematic review and meta-analysis for assessing the risk of *H. pylori* infection to overweight/obesity and examine mean difference in BMI with or without *H. pylori* infection by choosing subjects from different countries.

## 2 Methods

### 2.1 Study registration

The protocol for this systematic review and meta-analysis has been registered in the International Prospective Register of Systematic Reviews (PROSPERO), and the registration number is CRD42019121939. This systematic review and meta-analysis will be reported in accordance with the guidelines of the Cochrane Handbook for Systematic Reviews of Interventions and the PRISMA (Preferred Reporting Items for Systematic Reviews and Meta-Analyses) statement.^[24]^ Ethical approval is not required for this study.

### 2.2 Inclusion criteria for study selection

#### 2.2.1 Types of studies

Studies will included if they met all of the following inclusion criteria: original observational studies (including cross-sectional, case-control or longitudinal studies) that explored the association between *H. pylori* infection and overweight/obesity; studies reported *H. pylori* infection status in subjects with overweight/obesity and normal weight; outcome of interest is overweight/obesity; all studies that reported odds ratios (OR) or hazard ratios (HR) with 95% confidence intervals (CI) values for the exposure/outcome of interest; studies which overweight/obesity was diagnosed by BMI.

Criteria for exclusion were as follow: these studies, such as conference abstracts, case reports, case series, reviews, meta-analyses, practice guidelines, and comments, will be excluded; studies that did not specifically report any OR or HR and 95% CI for the outcome measure of interest; studies which were performed in patients with gastrointestinal cancer or treated with eradication drug therapy for *H. pylori*. As the included studies are observational in design, we will follow the Meta-analysis Of Observational Studies in Epidemiology (MOOSE) guidelines^[25]^ for the meta-analysis of the design, performance and reporting of observational studies.

#### 2.2.2 Types of population

Participants will be all subjects with and without *H. pylori* infection. There is no limitation on age, sex, and nationality.

#### 2.2.3 Types of exposures

*Helicobacter pylori* infection or positivity.

#### 2.2.4 Types of outcome measures

The primary outcome was the presence and incidence of overweight/obesity in conjunction with *H. pylori* infection.

### 2.3 Search strategy

We will extensively search the following 4 electronic bibliographic databases (PubMed, Web of science, The Cochrane Library, and EMBASE) for observational studies published until January 15, 2019 reporting the association between *H. pylori* infection and overweight/obesity or BMI. The search free text terms were “*Helicobacter pylori*” (OR “*Helicobacter pylori* infection” OR “*Helicobacter* infection” OR “Hp” OR “*H.pylori*” OR “*Campylobacter pylori*”) AND “obesity” OR “overweight” OR “body weight” OR “weight gain” OR “body mass index”. We also searched for MeSH (Medical Subject Headings) terms. Searches were restricted to human studies. Moreover, the reference lists in previously published reviews and original research articles will be scrutinized to identify publications not covered by the original database searches. No restrictions will be imposed regarding the language in which the studies were published. No filters were placed for the participants’ age, gender, race, and duration of disease. The search strategy for PubMed is shown in Table 1, other electronic databases will be searched based on this strategy.

**Table 1:**
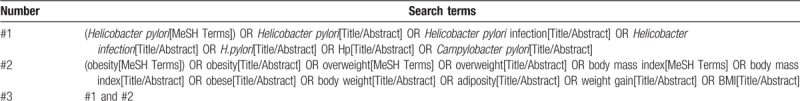
The search strategy for Pubmed.

### 2.4 Study selection and data extraction

#### 2.4.1 Data extraction and management

**Figure 1:**
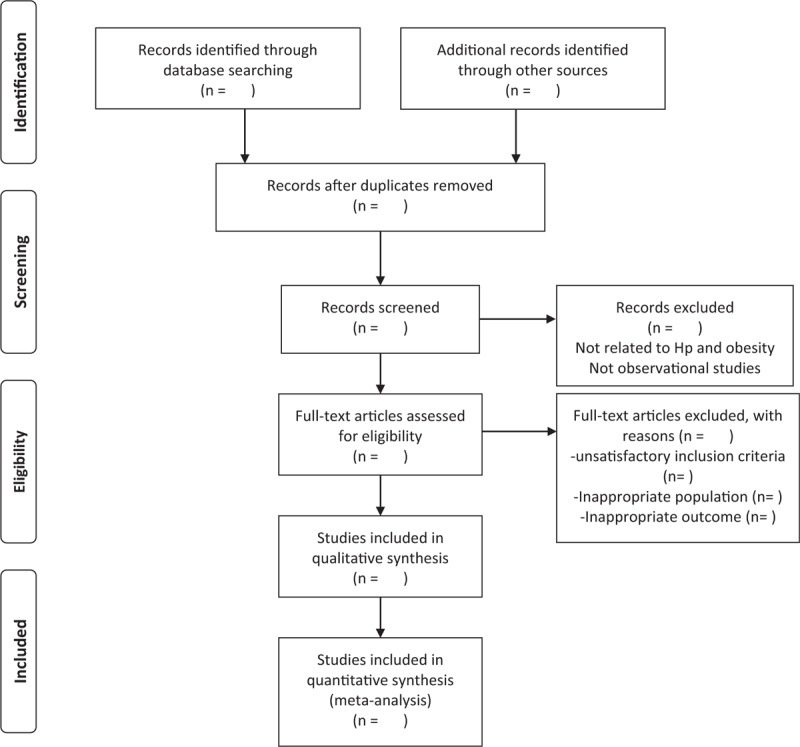
Flow diagram of study selection.

Two investigators (Jialing Chen and Jie Ma) independently examined all titles and abstracts, and obtained full texts of potentially relevant papers published until January 15, 2019. Any discrepancies will be resolved via a revaluation of the original article in consultation with a third author (Shukun Yao). The Flow diagram of study selection will be shown in Figure 1. For each study, the author list, country of origin, study design, mean age, sample size, sex ratio, year of publication, detection method used for the diagnosis of *H. pylori* infection, outcome of interest, follow-up duration, and list of covariates adjusted in multivariable analyses, will be extracted and recorded using a standard data extraction form.

#### 2.4.2 Assessment of risk of bias and study quality

Since all the included observational studies have a cross-sectional or case-control or cohort design, the Newcastle–Ottawa Quality Assessment Scale (NOS), which is a validated scale for non-randomized studies in meta-analyses, will be used to access the study quality by 2 review authors independently (Jialiang Chen and Jie Ma), as recommended by the Cochrane Collaboration.^[26]^ We judge studies that received a NOS score of at least 8 stars to be at low risk of bias, thus reflecting the highest quality. Any discrepancies will be addressed by a revaluation of the original article by a third author (Shukun Yao).

#### 2.4.3 Assessment of heterogeneity

We will start by looking at the forest plots for signs of heterogeneity. Statistical heterogeneity among studies will be assessed by the Chi-Squared test and the *I*^*2*^ statistic. When *I*^*2*^ < 50%, no significant heterogeneity will be concluded (or could be neglected). For these studies, a fixed effect model will be employed. For cases with significant heterogeneity (*I*^*2*^ > 50%), a random effect model will be employed. A Chi-Squared test *P* < .10 was used to determine statistical significance.

#### 2.4.4 Assessment of publishing bias

The possibility of publication bias will be evaluated using the funnel plot, Begg test and Egger regression test. We will assess publishing bias when 10 or more studies are included in this meta-analysis.

#### 2.4.5 Data synthesis

For dichotomous data, we calculated the odds ratio (OR) or HR and 95% CIs to express the effect size. For continuous data, we calculated mean differences (MD) with 95% CIs. For both individual and pooled data, we reported effect estimates from both continuous and dichotomous data as *P* values and 95% CI. We carried out statistical analyses using Review Manager 5.3 software and Stata 14.0 (StataCorp, College Station, TX). All tests were 2-tailed, and *P* < .05 was considered statistically significant.

#### 2.4.6 Subgroup analysis

If the necessary data are available, subgroup analyses will be carried out, if possible, for the impact of *H. pylori* infection on the risk of overweight/obesity, by study design, study country, the methods used for diagnosing *H. pylori* infection, levels of NOS, and whether they had full adjustment for covariates. Additionally, we tested for possibly excessive influence of individual studies using a meta-analysis influence test that eliminated each of the included studies at a time. Meta-regression analyses will be also performed.

#### 2.4.7 Sensitivity analysis

In the case of enough studies data, sensitivity analysis will be carried out to examine the robustness of the pooled results, regarding study characteristics and the methodological quality of the included studies. The effect on total effects will be examined by excluding some low-quality studies or small-sample sizes of studies. When heterogeneity exists, we may perform sensitivity analysis by excluding the data one by one.

## 3 Discussion

Currently, whether the relationship between *H. pylori* infection and overweight/obesity or BMI remains a controversy. Some published systematic review or meta-analyses did not combine data from the developed and developing countries. It is hard to judge the real role of *H. pylori* in overweight/obesity among overall population. Therefore, it is very necessary to reperform a systematic review and meta-analysis to investigate the relationship between them. We aim to summarize all evidence about published observational studies and provide evidence-based suggestions for *H. pylori* eradication treatment to prevent overweight/obesity. However, since those studies included in this systematic review will be all the observational studies, the results of this meta-analysis cannot establish a causal relationship between *H. pylori* infection and overweight/obesity.

## Author contributions

**Conceptualization:** Jialiang Chen, Jie Ma.

**Data curation:** Jialiang Chen, Jie Ma.

**Formal analysis:** Jialiang Chen, Jie Ma, Shaojie Duan.

**Funding acquisition:** Shukun Yao.

**Methodology:** Xinyuan Liu, Ning Liang.

**Supervision:** Shukun Yao.

**Writing – original draft:** Jialiang Chen, Jie Ma.

**Writing – review & editing:** Jialiang Chen, Shukun Yao.

Shukun Yao orcid: 0000-0002-4826-5169.
